# Coronary artery spasms and/or coronary air embolisms and contrast-induced encephalopathy during cryoablation of atrial fibrillation: a case report

**DOI:** 10.1093/ehjcr/ytaf413

**Published:** 2025-08-22

**Authors:** Togo Sakai, Masao Takemoto, Taisuke Kitamura, Takuya Tsuchihashi

**Affiliations:** Cardiovascular Centre, Social Medical Corporation Steel Memorial Yawata Hospital, 1-1-1 Haruno-machi, Yahatahigashi-ku, Kitakyushu 805-8508, Japan; Cardiovascular Centre, Social Medical Corporation Steel Memorial Yawata Hospital, 1-1-1 Haruno-machi, Yahatahigashi-ku, Kitakyushu 805-8508, Japan; Cerebrovascular Centre, Social Medical Corporation Steel Memorial Yawata Hospital, 1-1-1 Haruno-machi, Yahatahigashi-ku, Kitakyushu, Japan; Cardiovascular Centre, Social Medical Corporation Steel Memorial Yawata Hospital, 1-1-1 Haruno-machi, Yahatahigashi-ku, Kitakyushu 805-8508, Japan

**Keywords:** Ablation, Atrial fibrillation, Case report, Computed tomography, Contrast-induced encephalopathy, Coronary artery spasm, Coronary air embolism

## Abstract

**Background:**

ST-segment elevations in electrocardiogram associated with coronary air embolisms (CAEs) and/or myocardial ischaemia induced by coronary artery spasms (CASs) are rare complications during ablation of atrial fibrillation (AF); some patients develop severe conditions. Contrast-induced encephalopathy (CIE) is also a rare but severe complication associated with the use of iodinated contrast agents during various cardiovascular and neurovascular procedures. Its occurrence during ablation, particularly AF ablation, remains unreported.

**Case summary:**

We report a case in which CASs and/or CAEs associated with ST-segment elevation were observed, accompanied by worsening haemodynamics during AF cryoablation. Insertion of an intra-aortic balloon pump improved the haemodynamics. Following anaesthesia recovery, he developed verbal/expressive aphagia and left-sided hemiplegia. However, emergent cerebrovascular angiography revealed no cerebral artery occlusions or haemorrhage. Plain computed tomography (CT) showed diffuse cortical hyperdensity in the right temporal and both occipital lobes on Day 0, brain swelling by Day 2, and complete radiological recovery by Day 4. His symptoms, except for a slight grip drop of the left hand, improved by Day 4, leading to a diagnosis of CIE.

**Discussion:**

The key step of CIE may be attributed to a blood–brain barrier breakdown, which is influenced by multiple factors, including the direct chemotoxicity and hyperosmolarity of the contrast agent, ischaemic stroke (including cerebral vasoconstriction), and anaesthesia. Thus, physicians should consider the possibility of CIE when a patient exhibits neurological abnormalities during ablation, despite a lack of a CIE history with contrast-enhanced CT.

Learning points

- Coronary artery spasms (CASs) and/or coronary air embolisms (CAEs) and contrast-induced encephalopathy (CIE) are rare complications during various cardiovascular and neurovascular procedures, but both might occur at the same time. It may be sometimes difficult to distinguish between CASs and CAEs during ablation.
- Physicians should consider the possibility of CIE when a patient exhibits neurological abnormalities during/after ablation, even though there is no history of CIE with contrast-enhanced computed tomography.
- Understanding the pathogenesis and risk factors for CIE in this context is critical for improving patient safety and outcomes. Further investigation is required to increase clarity surrounding this poorly understood CIE mechanism.

## Introduction

ST-segment elevation in the electrocardiogram associated with coronary air embolisms (CAEs) and/or myocardial ischaemia induced by coronary artery spasms (CASs) is a rare complication during ablation of atrial fibrillation (AF); some patients develop severe conditions.^1–3^ Contrast-induced encephalopathy (CIE) is also a rare but severe complication associated with the use of iodinated contrast agents during various cardiovascular and neurovascular procedures.^4,5^ Its occurrence during ablation, particularly AF ablation, remains unreported. Herein, we report a case of both ST-segment elevations in the electrocardiogram associated with CAEs and/or CASs and CIE simultaneously during/after AF ablation.

## Summary figure

**Figure:**
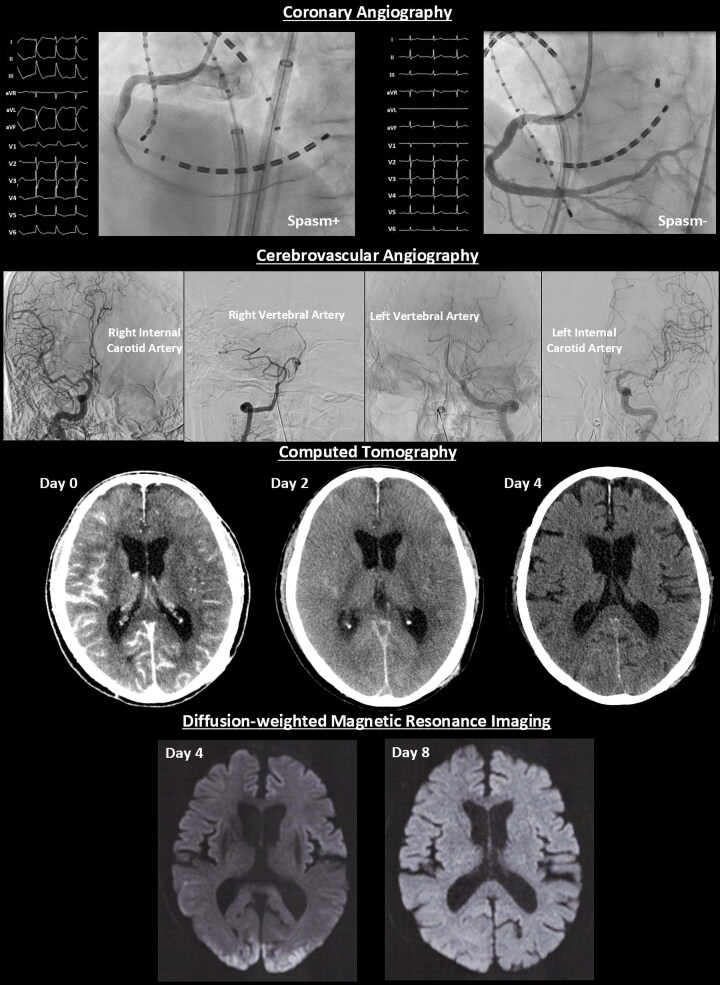


## Case presentation

**Figure 1:**
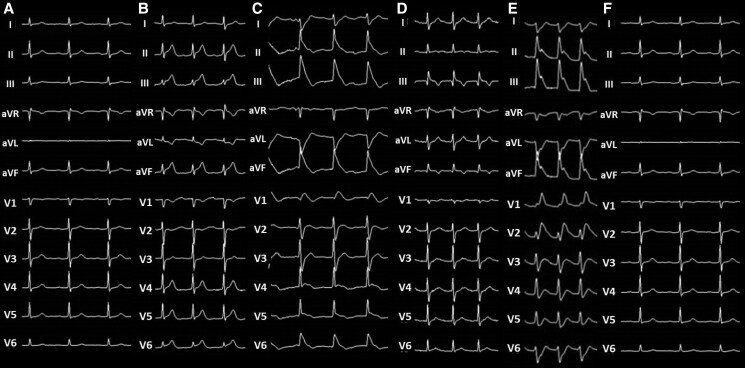
Twelve-lead electrocardiograms before ablation (*A*), after the transseptal puncture (*B* and *C*), and after intra-aortic balloon pump insertion (*D*).

**Figure 2:**
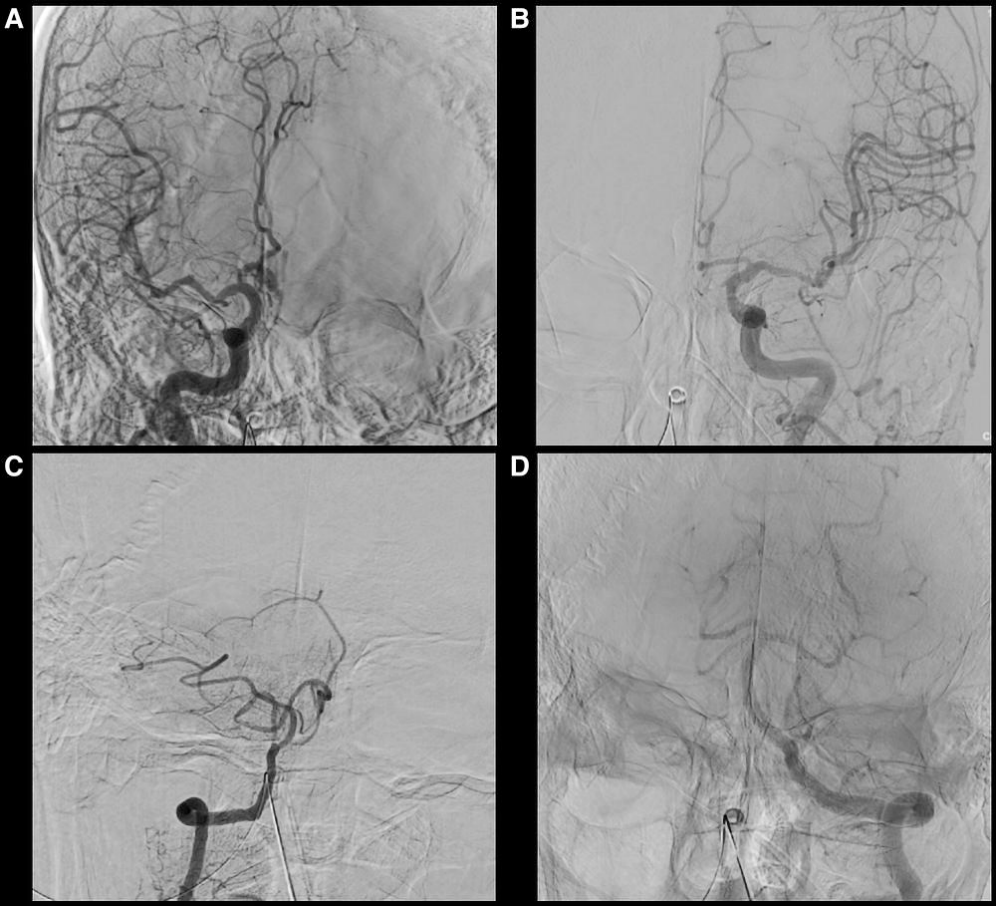
Emergent cerebrovascular angiography of the right (*A* and *C*) and left (*B* and *D*) internal carotid arteries (*A* and *B*) and vertebral artery (*C* and *D*).

**Figure 3:**
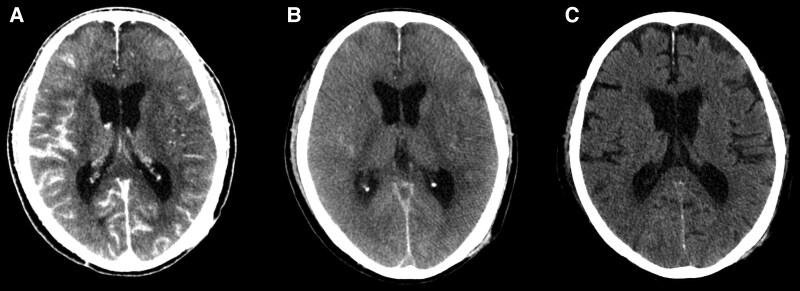
Plain head computed tomography on Days 0 (*A*), 2 (*B*), and 4 (*C*).

**Figure 4:**
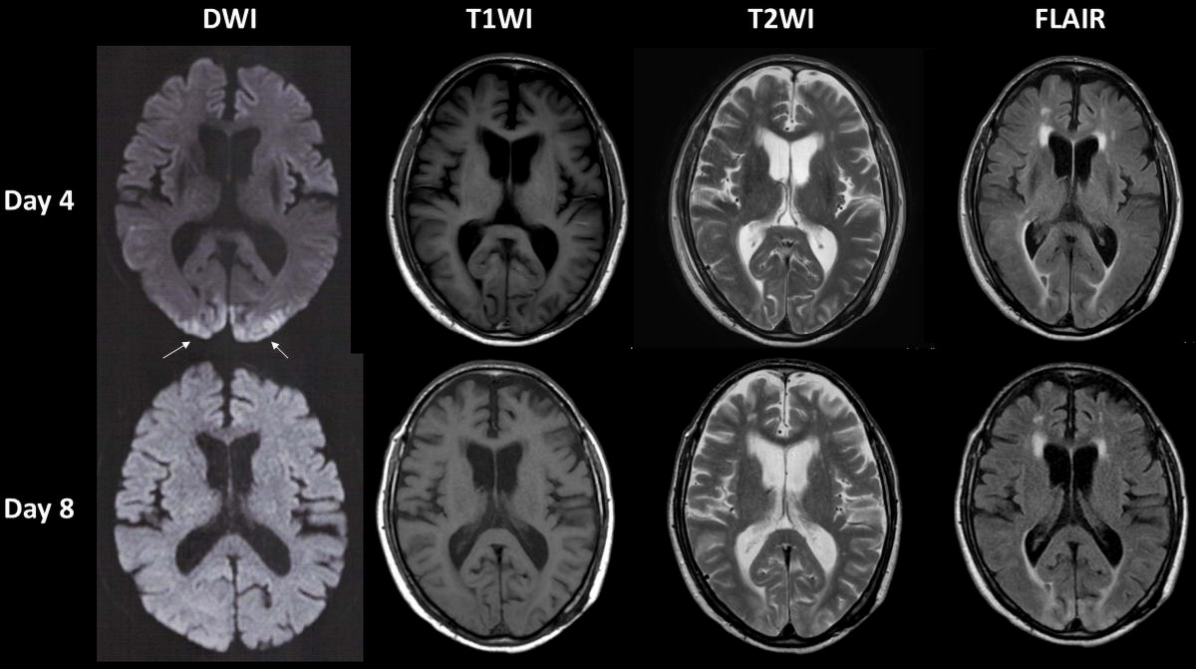
Diffusion-, T1-, and T2-weighted and fluid-attenuated inversion recovery images obtained via magnetic resonance imaging of the head on Days 4 and 8.

An 80-year-old man with a chief complaint of palpitations with a history of hypertension, dyslipidaemia, and chronic kidney disease was admitted to undergo AF cryoablation. He received optimized medical therapy, including edoxaban 30 mg, bepridil 100 mg, olmesartan 20 mg, amlodipine 5 mg, carvedilol 10 mg, and rosuvastatin 2.5 mg once daily. On admission, he had a blood pressure of 124/66 mmHg and a regular heart rate of 60 b.p.m. Precordial auscultation revealed normal cardiac and respiratory sounds. His body mass index was 20.2 kg/m^2^, and his estimated glomerular filtration rate and levels of serum creatinine and LDL and HDL cholesterol were 36.3 mL/min/1.73m^2^ and 1.45, 70 mg/dL and 55 mg/dL, respectively. A 12-lead electrocardiogram revealed sinus rhythm (*Figure 1A*). Echocardiography revealed a normal left ventricular ejection fraction and left atrial (LA) enlargement of 38 mm. The patient’s CHADS_2_/CHA_2_DS_2_-VASc score was 2/3. Before the procedure, contrast-enhanced (Iopamidol™, Fuji Pharma Co., Ltd., Japan) cardiac computed tomography (CT) revealed a normal anatomy of the LA and PVs. After anaesthesia/sedation induction with propofol and an intravenous administration of dexmedetomidine, a 15-Fr cryo-sheath was placed into the left atrium following a single transseptal puncture. Subsequently, the 12-lead electrocardiogram demonstrated ST-segment elevation in leads II, III, aVF, and V5–6 (*Figure 1B* and *C*). Emergent coronary angiography (Iopamidol™) revealed coronary slow flow (CSF) without significant stenosis in the right coronary artery (RCA) (see Supplementary material online, *Video S1A*), whereas the left coronary artery appeared normal. Intracoronary isosorbide dinitrate (ISDN) administration steadily improved the ST-segment elevation (*Figure 1D*) associated with the CSF (see Supplementary material online, *Video S1B*). However, the ST-segment elevation associated with the CSF recurred (*Figure 1E*), despite repeated intracoronary ISDN administration, accompanied by gradual worsening haemodynamics. Intra-aortic balloon pumping (IABP) was immediately performed to diagnose CAEs and/or drug-refractory microvascular CASs.^6,7^ Subsequently, the haemodynamics and ST-segment elevation steadily improved (*Figure 1F*). Upon recovery from anaesthesia, he developed verbal/expressive aphagia and left-sided hemiplegia. Therefore, we suspected that this outcome was caused by an occlusion of a cerebral artery, which could have resulted in a cerebral infarction; this occlusion could have also included an air embolism or haemorrhage driven by an aneurysm of a cerebral artery. However, emergent cerebrovascular angiography (Iopamidol™) revealed no evidence of a cerebral artery occlusion or haemorrhage (*Figure 2A–D*). Plain CT on Day 0 showed diffuse cortical hyperdensity in the right temporal and both occipital lobes (*Figure 3A*). By Day 2, brain swelling occurred (*Figure 3B*), with complete radiological recovery by Day 4 (*Figure 3C*). After withdrawal of the IABP, diffusion-weighted magnetic resonance imaging (MRI) on Day 4 revealed diffusion restrictions of both occipital lobes (white arrows in *Figure 4*), indicating brain oedema, with complete recovery by Day 8 (*Figure 4*). His symptoms except for a slight grip drop of the left hand improved by Day 4, leading to a diagnosis of CIE. He was discharged after rehabilitation and has remained well without AF attacks under the internal use of bepridil 50 mg for more than 2 years after this event.

## Discussion

The incidence of CAS-associated AF ablation is 0.19%–0.31%, and one-sixth of patients with CASs have a severe condition.^1,2^ The presumed major mechanism underlying CASs includes a change in autonomic tone associated with epicardial ganglionated plexi through thermal or cooling injury, which causes a sudden increase in the sympathetic or vagal tone and triggers CASs.^1,2^ In this present case, however, the CASs occurred just after the transseptal puncture. The atrial septum is concentrated with a high density of parasympathetic fibres, which preferentially innervate the RCA, leaving it vulnerable to cholinergic vasospasms.^8^ In addition, the procedure was performed under intravenous administration of dexmedetomidine, which stimulates α2-adrenergic receptors, and strained vagal tone induces CASs.^9^ Thus, the passage of the catheter through these high-density nerve complexes of the atrial septum during the transseptal puncture and the effect of dexmedetomidine might cause CASs of the RCA. Although the incidence of CAE-associated cryoablation is 2.6%, all cases recovered under conservative treatment without any sequela.^3^ Coronary air embolisms during ablation usually occur when the inner sheath is taken out.^10^ In this present case, ST-segment elevation on the electrocardiogram occurred before the sheath was taken out. Actually, the cryo-inner sheath could be seen during ST-segment elevation (see Supplementary material online, *Video S1A*). It may sometimes be difficult to distinguish between CASs and CAEs during ablation. Coronary artery spasms may usually be reversed by an intracoronary ISDN administration. However, some cases have been reported to have CASs refractory to drugs, including ISDN,^6,7^ as in this case. On the contrary, a recent study showed that all patients with CAEs recovered under conservative treatment.^3^ However, a recent case report demonstrated that CAEs became catastrophic during cryoablation.^11^ This present case also developed a severe condition, and IABP was needed to improve his haemodynamics and ST-segment elevation associated with CSF. It might be difficult to make a definite diagnosis of CASs or CAEs in this present case.

Basically, there are no specific imaging findings for CIE. Thus, it is a diagnosis of exclusion. Since the duration of the worsening haemodynamics was about a few minutes, global cerebral hypoperfusion might have been unlikely. Plain CT on Day 0 showed diffuse cortical hyperdensity in the right temporal and both occipital lobes involving the subarachnoid space (*Figure 3A*). Thus, we had to consider the possibility of a subarachnoid haemorrhage. However, emergent cerebrovascular angiography revealed no evidence of a cerebral artery occlusion, including air embolisms or haemorrhages (*Figure 2A–D*) driven by ruptured aneurysm(s) of a cerebral artery. Hence, a cerebral infarction or haemorrhage might also have been unlikely. Diffusion-weighted MRI on Day 4 revealed diffusion restrictions of both occipital lobes (white arrows in *Figure 4*). Those findings pointed to the possibility of posterior reversible encephalopathy syndrome (PRES). However, the absence of a headache, seizures, altered mental status, visual loss, and high blood pressure in the present case did not allow for a definitive PRES diagnosis. Finally, his radiological findings and symptoms, except for a slight grip drop of the left hand, improved by Day 4, leading to a diagnosis of CIE.

The incidence of CIE is 0.05%–0.4% or 1.7%–3.6% following coronary or cerebral angiography and interventions.^4^ However, CIE occurrence during ablation procedures remains unreported. Although the mechanism of CIE has not been entirely elucidated, the pathogenesis is multifocal.^4^ The key step of CIE may be attributed to a blood–brain barrier (BBB) breakdown, influenced by multiple factors, including direct chemotoxicity and hyperosmolarity of the contrast agent, ischaemic stroke and/or vasoconstriction,^4^ hypertension, chronic kidney disease, and anaesthesia.^5^ In this present case, an ischaemic stroke might have been caused by a cerebral air embolism,^12^ but a CIE occurrence associated with a cerebral air embolism remains unreported. Different types (high-/low-osmolality, ionic, or non-ionic) and dosages of contrast media can lead to CIE. In this case, CIE occurred only during ablation despite using the same contrast medium, Iopamidol™, during pre-procedural contrast-enhanced CT, coronary angiography, and cerebrovascular angiography. Thus, a multifactorial hypothesis may be supported. However, since the dosage of the contrast media was higher for coronary angiography (75 mL) and cerebrovascular angiography (60 mL) than for pre-procedural contrast-enhanced CT (80 mL), it might have at least affected the occurrence of CIE. The cerebrovascular angiography (*Figure 2A–D*), head CTs (*Figure 3A–C*), and MRIs (*Figure 3D* and *E*) revealed no evidence of a cerebral artery occlusion/infarction or haemorrhage. Because CASs are infrequently linked to cerebral vasoconstriction and neurological sequelae,^4^ and dexmedetomidine stimulates α2-adrenergic receptors, cerebral vasoconstriction contributing to an ischaemic stroke before cerebrovascular angiography might occur. A recent report has shown that general anaesthesia may be one of the critical risk factors of CIE.^5^ Moreover, this report mentioned that propofol, used in this case, might have been associated with the increased risk of CIE because it was observed to provide a better disruption of the BBB.^5^ Once across the BBB, contrast media may exert neurotoxic effects and lead to cerebral oedema (*Figure 3B* and *D*), due to changes in the oncotic pressure gradients. Further investigation is required to increase the clarity surrounding this poorly understood mechanism of CIE.

## Conclusions

Physicians should consider the possibility of CIE when a patient exhibits neurological abnormalities during/after ablation, even though there is no history of CIE with contrast-enhanced CT.

## Lead author biography


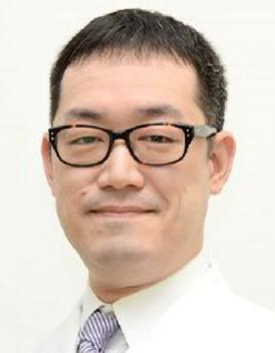


Dr Togo Sakai received his MD degree from Saga University (Saga, Japan) in 2013. Since 2020, he has been working as a cardiologist at the Social Medical Corporation Steel Memorial Yawata Hospital. His area of medical interest is heart failure, arrhythmias, sleep disorder, cardiac rehabilitation, and coronary artery disease.

## Supplementary Material

ytaf413_Supplementary_Data

## Data Availability

The de-identified participant data will not be shared.

## Notes

**Consent:** The authors confirm that the patient has given written consent for submitting and publishing this case report, including the image(s) and associated text, per COPE guidance.

**Funding:** None declared.
